# First records of the *Palaestes abruptus* Sharp, 1899 and *P. nicaraguae* Sharp, 1899 (Coleoptera: Cucujidae) from South America, with a checklist of flat bark beetles from the continent

**DOI:** 10.3897/BDJ.9.e62576

**Published:** 2021-02-17

**Authors:** Radomir Jaskuła, Marek Michalski, John W. M. Marris

**Affiliations:** 1 Department of Invertebrate Zoology and Hydrobiology, Faculty of Biology and Environmental Protection, University of Lodz, Łódź, Poland Department of Invertebrate Zoology and Hydrobiology, Faculty of Biology and Environmental Protection, University of Lodz Łódź Poland; 2 Department of Experimental Zoology and Evolutionary Biology, Faculty of Biology and Environmental Protection, University of Lodz, Łódź, Poland Department of Experimental Zoology and Evolutionary Biology, Faculty of Biology and Environmental Protection, University of Lodz Łódź Poland; 3 Entomology Research Collection, Bio-Protection Research Centre, Lincoln University, Christchurch, New Zealand Entomology Research Collection, Bio-Protection Research Centre, Lincoln University Christchurch New Zealand

**Keywords:** *
Palaestes
*, *
Thesaurus
*, Cucujidae, Coleoptera, Brazil, Ecuador, Peru, Venezuela, checklist, iNaturalist, citizen science

## Abstract

**Background:**

The flat bark beetles (Coleoptera: Cucujidae) is a small insect family with only about 70 species. Most of the species are distributed in Holarctic, Oriental and/or Australasian realms, while in South America, only six species have been recorded, including a single one known from Peru.

**New information:**

Two cucujid beetle species, *Palaestes abruptus* Sharp, 1899 and *P. nicaraguae* Sharp, 1899, are recorded from South America for the first time. The species are recorded from the Pasco (*P. abruptus*) and Cusco and Junín (*P. nicaraguae*) Regions of Peru, based, in part, on data collected through the iNaturalist citizen science database. Habitats of both species are presented in photographs for the first time. A country-level checklist to Cucujidae species currently known from South America is provided.

## Introduction

The flat bark beetles (Coleoptera: Cucujidae) form a small insect family which includes five genera and almost 70 species and is distributed worldwide, except for Africa, Antarctica, most of the oceanic islands and the greater part of the Arctic (Thomas 2003, Lee and Satô 2007, Lee and Pütz 2008, Horák and Chobot 2009, Lee and Thomas 2011, Bonacci et al. 2012, Marris and Ślipiński 2014, Bussler 2017, Marris 2017, Háva et al. 2019, Zhao and Zhang 2019, Hsiao 2020, Jaskuła et al. 2020, Jin et al. 2020). The South American cucujid fauna is represented by two genera belonging to the subfamily Platisinae: *Palaestes* Perty, 1830 and *Thesaurus* Jin, Zwick, Ślipiński, Marris, Thomas & Pang 2020. Until now, only one species, *Thesaurus zaitsevi* Jin, Zwick, Ślipiński, Marris, Thomas & Pang 2020 has been confirmed from Peru (Jin et al. 2020).

This paper presents the first confirmed occurrence of the genus *Palaestes* Perty, 1830 from Peru, based on records of *Palaestes abruptus* Sharp, 1899 and *P. nicaraguae* Sharp, 1899. These are also the first records of these species from South America. A checklist of the flat bark beetle species, known from South America, is given.

## Materials and methods

South America ranks as the fourth largest continent, with an area of almost 18 million square kilometres. Brazil is the largest country, encompassing around half of the continent's land area. The greater part of northern and central regions of South America (much of Brazil, Colombia, Ecuador, Guyana, French Guiana, Suriname, most of Peru, north-eastern Paraguay and Venezuela) fall within the tropical and subtropical climatic zones, with a mainly temperate climate in the southern regions. The western part of the continent is characteristically arid (areas in Chile, Peru, Argentina and Paraguay), while a cold, polar climate is present only in southern regions of Argentina and Chile (Beck et al. 2018). As a consequence of its climatic conditions and diverse topography, South America is one of the most biodiverse continents on Earth with thousands of endemic species (Myers et al. 2000).

This project was prompted from viewing a single male *Palaestes abruptus* from Peru (Pasco Region, Oxapampa Province, Pozuzo District, 5 km E Santa Rosa, 10.0006S, 75.479W, 1150 m a.s.l., 25.11.2016) in a photo published on the iNaturalist website (www.inaturalist.org). The iNaturalist site allows observers from around the world to post images of organisms on the website, while others provide identifications. Although the observer of the *Palaestes abruptus* photograph (www.inaturalist.org/observations/36220768) was unable to send the material for study, he provided information that allowed RJ to contact Mr. Pavel Udovichenko, an insect dealer in Russia, who had additional material of this species. Subsequently, two male specimens were received from this seller. Moreover, Mr. Andrey Azarov sent us a pair of *Palaestes nicaraguae* adults for study. These specimens are currently deposited in the first author’s collection (RJC). Three further specimens from Peru were examined by JM from loan material from the Florida State Collection of Arthropods, Gainesville, Florida, USA (FSCA), provided by the late Michael Thomas.

The specimens examined in this study were identified, based on the original species descriptions (Sharp 1899) and by comparison with photographs of type material.

## Taxon treatments

### Palaestes abruptus

Sharp, 1899

64FD53F4-FE8A-5EB8-8A94-53CE3A5B658E

#### Materials

- **Type status:** Other material. **Occurrence:** sex: 2 males; lifeStage: adult; **Taxon:** higherClassification: Animalia; Arthropoda; Insecta; Coleoptera; Cucujidae' Palaestes; kingdom: Animalia; phylum: Arthropoda; class: Insecta; order: Coleoptera; family: Cucujidae; genus: Palaestes; specificEpithet: abruptus; **Location:** higherGeography: South America; Peru; Pasco Region; Oxapampa Province; Pozuzo District; Pozuzo; continent: South America; country: Peru; countryCode: PE; stateProvince: Pasco Region; county: Oxapampa Province; municipality: Pozuzo District; locality: Pozuzo; verbatimElevation: 750-1430 m; **Identification:** identifiedBy: Radomir Jaskuła; dateIdentified: 2020-12; identificationReferences: Sharp 1899; identificationRemarks: Pictures of type material were used to confirm identification; **Event:** eventDate: 2019-11; year: 2019; month: 11; habitat: mountain rain forest; eventRemarks: col. Alexander Sokolov; **Record Level:** collectionID: RJC (Radomir Jaskuła Collection, Łódź, Poland); ownerInstitutionCode: RJC/CUC-0008; RJC/CUC-0009; basisOfRecord: PreservedSpecimen

#### Description

**Figure 1.**
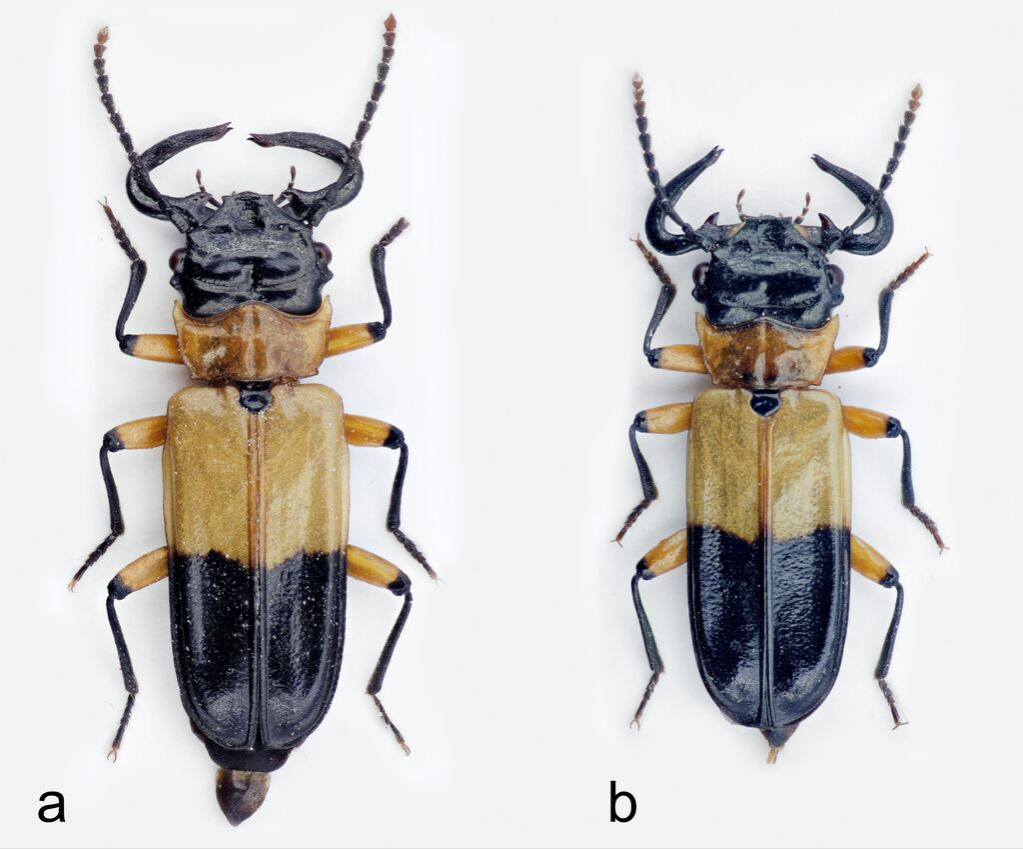
*Palaestes abruptus* – two males (a-b) from Pasco Region, Peru (photo Marek Michalski).

Small beetles with strongly flattened bodies; body length (measured from the top of clypeus to the end of elytra) of two studied Peruvian males: 14.7 mm (Fig. 1a) and 13.6 mm (Fig. 1b). Head black with mandibles black; pronotum yellow-orange; basal part of elytra yellow-orange, while the second half is black; scutellum black; antennae black, except for the last two antennomeres which are brown-orange; legs yellow-orange, except apical parts of femur, tibia and tarsus which are black; tarsal claws brown-orange. Sexual dimorphism very visible with males having very large mandibles.

#### Distribution

**Figure 2.**
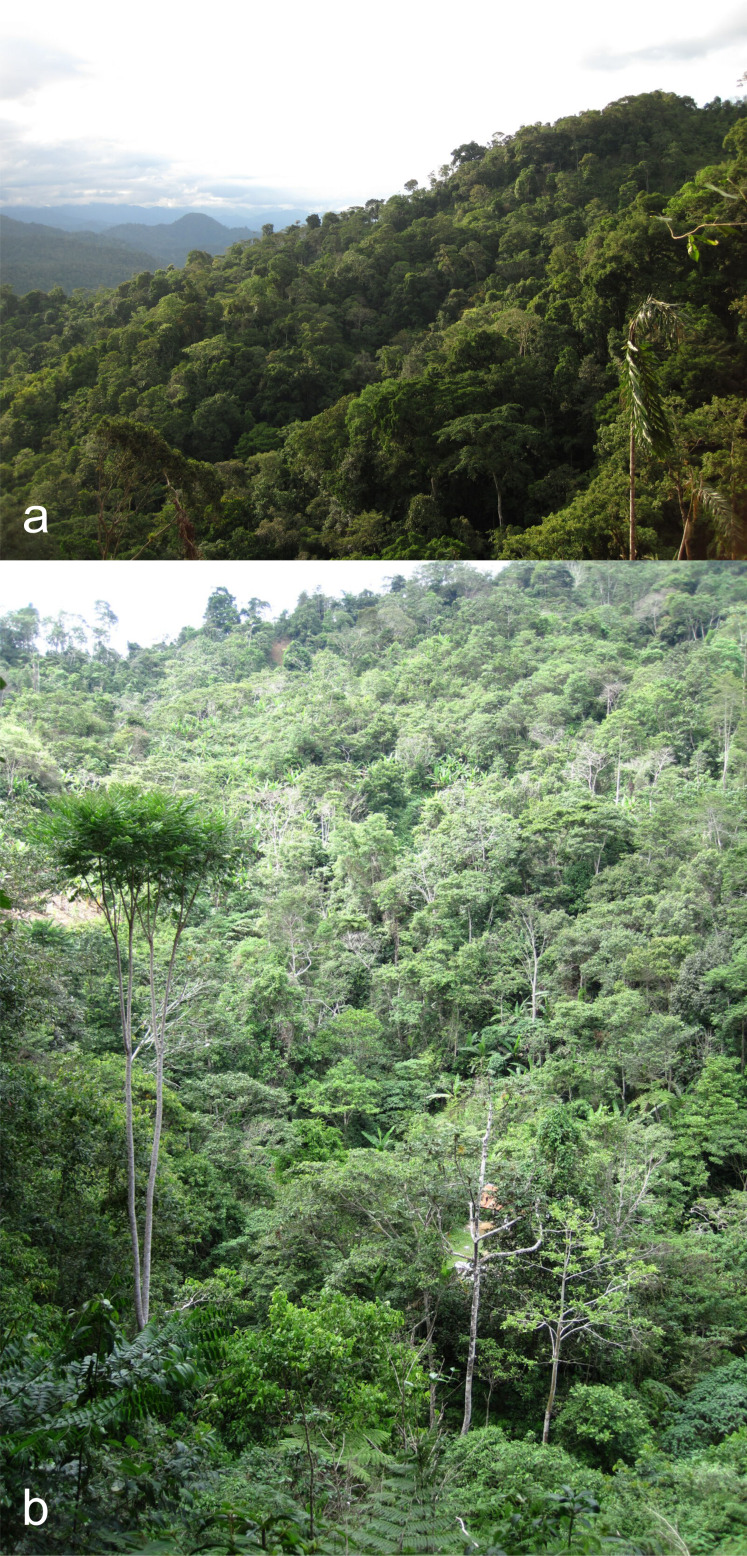
Habitat of *Palaestes abruptus* in Pasco Region, Peru: general view of the mountains (a), rain forest (b) (photo Alexander Sokolov).

Previously, this species was only known from Panama and Costa Rica (Sharp 1899, Jin et al. 2020). The locality is mountainous rain forest at altitudes ranging from 750 to 1430 m (Fig. 2). This is the first record from Peru and South America.

#### Ecology

Cucujidae larvae and adults are known to live under the bark of dead trees (Thomas and Leschen 2010); however, very little is known about biology and ecology of the *Palaestes* species. Sharp (1899) reported that “Mr Champion informs me that these insects are chiefly found between the thin crevices of sappy timber, and that they are often seen on the wing in forest clearings”. This indicates that the habit of *Palaestes* is similar to that of other cucujids. The only published account of the biology specifically for *P. abruptus* was by Jin et al. (2020), who recorded the only known larva of the species from a rotten log in Costa Rica, found in association with an adult female. *Palaestes* larvae and adults are probably predatory on small invertebrates living under the bark of dead trees, as has been noted for *Platisus zelandicus* Marris & Klimaszewski, 2001 (Watt et al. 2001) and *Cucujus* spp. (e.g. Palm 1941, Mamaev et al. 1977, Smith and Sears 1982, Horák and Nakládal 2009, Mazzei et al. 2011, Bonacci et al. 2012, Bonacci et al. 2020, Zdeněk et al. 2012). However, it cannot be excluded that they feed also as scavengers or as opportunistic omnivores, feeding on various types of organic debris, such as wood and phloem debris, as recorded for some *Cucujus* species (Nikitskiy et al. 2000, Horák and Nakládal 2009, Horák 2011). Detailed studies of the phenology, food and habitat preferences and behaviour of *Palaestes* species are needed.

### Palaestes nicaraguae

Sharp, 1899

FB27626B-98A1-50B0-9EBF-8A8A5ED34E1C

#### Materials

- **Type status:** Other material. **Occurrence:** sex: 1 male, 1 female; lifeStage: adult; **Taxon:** higherClassification: Animalia; Arthropoda; Insecta; Coleoptera; Cucujidae' Palaestes; kingdom: Animalia; phylum: Arthropoda; class: Insecta; order: Coleoptera; family: Cucujidae; genus: Palaestes; specificEpithet: nicaraguae; **Location:** higherGeography: South America; Peru; Junín Region; Satipo Province; Satipo District; 15 km N-NW from Satipo; Rio Venado vill.; continent: South America; country: Peru; countryCode: PE; stateProvince: Junín Region; county: Satipo Province; municipality: Satipo District; locality: Rio Venado vill., 15 km N-NW from Satipo; verbatimElevation: 1300 m; verbatimCoordinates: 11°11.32S 74°46.03W; **Identification:** identifiedBy: Radomir Jaskuła; dateIdentified: 2020-12; identificationReferences: Sharp 1899; identificationRemarks: Pictures of type material were used to confirm identification; **Event:** eventDate: 2019-11-01; year: 2019; month: 11; day: 1; habitat: mountain rain forest; eventRemarks: col. Alexander Petrov; **Record Level:** collectionID: RJC (Radomir Jaskuła Collection, Łódź, Poland); ownerInstitutionCode: RJC/CUC-0010; RJC/CUC-0011; basisOfRecord: PreservedSpecimen
- **Type status:** Other material. **Occurrence:** sex: male; lifeStage: adult; associatedOccurrences: NCBI BioSample ID: SAMN10963593; associatedSequences: GenBank: No. MK614522; **Taxon:** kingdom: Animalia; phylum: Arthropoda; class: Insecta; order: Coleoptera; family: Cucujidae; genus: Palaestes; specificEpithet: nicaraguae; **Location:** higherGeography: South America; Peru; Cusco Region; Sancurtambo; Santa Isabel; Cosnipata [Kosñipata] Valley Rain Forest Alt. 5525 ft.; continent: South America; country: Peru; countryCode: PE; stateProvince: Cusco Region; locality: Cosnipata [Kosñipata] Valley Rain Forest Alt. 5525 ft.; **Identification:** identifiedBy: Mengjie Jin; dateIdentified: 2019; identificationReferences: Sharp 1899; identificationRemarks: Pictures of type material were used to confirm identification; **Event:** eventDate: 1951-12-14; year: 1951; month: 12; day: 14; eventRemarks: col. F Woytkowski; **Record Level:** collectionID: FSCA (Florida State Collection of Arthropods, Gainesville, Florida, USA); basisOfRecord: PreservedSpecimen
- **Type status:** Other material. **Occurrence:** sex: male; lifeStage: adult; **Taxon:** higherClassification: Animalia; Arthropoda; Insecta; Coleoptera; Cucujidae' Palaestes; kingdom: Animalia; phylum: Arthropoda; class: Insecta; order: Coleoptera; family: Cucujidae; genus: Palaestes; specificEpithet: nicaraguae; **Location:** higherGeography: South America; Peru; Cusco Region; Sancurtambo; Santa Isabel; Cosnipata [Kosñipata] Valley Rain Forest Alt. 5525 ft.; continent: South America; country: Peru; countryCode: PE; stateProvince: Cusco Region; locality: Cosnipata [Kosñipata] Valley Rain Forest Alt. 5525 ft.; **Identification:** identifiedBy: Mengjie Jin; dateIdentified: 2019; identificationReferences: Sharp 1899; identificationRemarks: Pictures of type material were used to confirm identification; **Event:** eventDate: 1951-12-14; year: 1951; month: 12; day: 14; eventRemarks: col. H. L. Dozier; **Record Level:** collectionID: FSCA (Florida State Collection of Arthropods, Gainesville, Florida, USA); basisOfRecord: PreservedSpecime
- **Type status:** Other material. **Occurrence:** sex: female [genitalia dissected]; lifeStage: adult; **Taxon:** higherClassification: Animalia; Arthropoda; Insecta; Coleoptera; Cucujidae' Palaestes; kingdom: Animalia; phylum: Arthropoda; class: Insecta; order: Coleoptera; family: Cucujidae; genus: Palaestes; specificEpithet: nicaraguae; **Location:** higherGeography: South America; Peru; Cusco Region; Quiroz; Rio Paucartambo; continent: South America; country: Peru; countryCode: PE; stateProvince: Cusco Region; locality: Quiroz, Rio Paucartambo; **Identification:** identifiedBy: John W.M. Marris; dateIdentified: 2020-12; identificationReferences: Sharp 1899; identificationRemarks: Pictures of type material were used to confirm identification; **Event:** eventDate: 1933-11-23; year: 1933; month: 11; day: 23; **Record Level:** collectionID: FSCA (Florida State Collection of Arthropods, Gainesville, Florida, USA); basisOfRecord: PreservedSpecimen

#### Description

**Figure 3.**
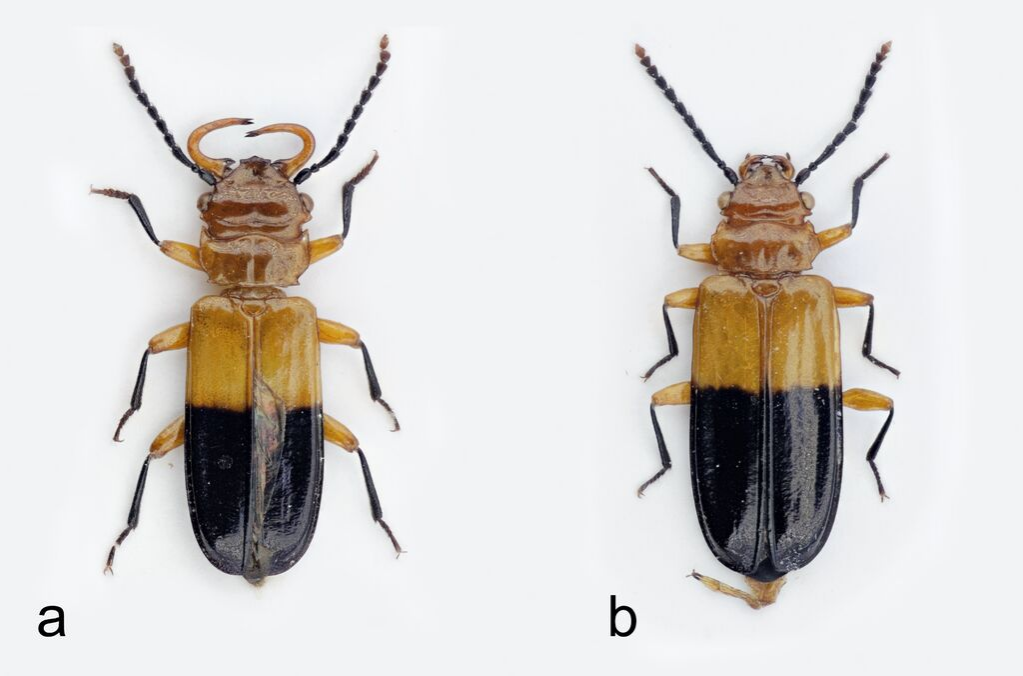
*Palaestes nicaraguae* – male (a) and female (b) from Junín Region, Peru (photo Marek Michalski).

Small beetles with strongly flattened bodies; body length (measured from the top of clypeus to the end of elytra) from 10.2 mm (female, Fig. 3b) to 10.4 mm (male, Fig. 3a). Sexual dimorphism clearly visible with males having very large mandibles.

The Peruvian specimens match closely with the holotype of *P. nicaraguae*, based on examination of photographs of the holotype (held in the Natural History Museum, London). The specimens from Peru share the following distinctive characteristics: head, mandibles and prothorax orange-brown, scutellum orange-brown, legs with femorae orange-brown and tibiae and tarsi black, tarsal claws brown-orange and elytra with black colouration extending apically from about the basal 1/3 to 2/5. The only notable difference is that, in the specimens from Peru, the antennomeres dark brown to black, except for the last two apical segmenta, which are red-brown. In contrast, the holotype has three apical segments that are red-brown.

#### Distribution

**Figure 4.**
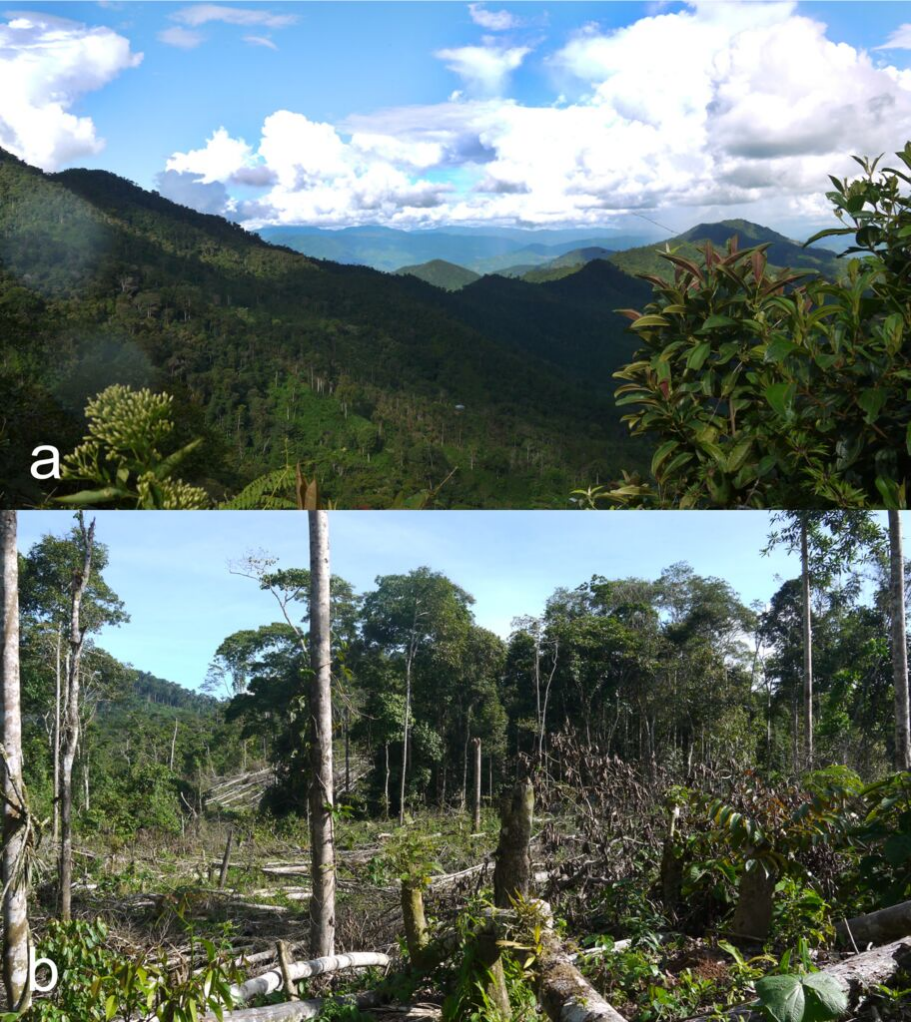
Habitat of *Palaestes nicaraguae* in Junín Region, Peru: general view (a), felled trees (b) (photo Alexander Petrov).

Previously, this species was known only from Nicaragua (Sharp 1899, Jin et al. 2020). The locality is placed in mountainous rain forest at altitudes from 1100 to 1350 m a.s.l. (Fig. 4). These are the first records from Peru and South America.

#### Ecology

As noted for *P. abruptus*, the larvae and adults of *P. nicarague* are presumed to live under the bark of dead trees, where they most probably prey on small insects and larvae. There are no published accounts of the ecology and biology specifically for *P. nicaraguae*.

## Discussion

The flat bark beetle fauna (Coleoptera: Cucujidae) of South America has never been intensively studied and only a few papers have focused on the insect family in South America. For *Palaestes* Perty, 1830, original species descriptions were provided by von Heyden (1827) for *P. freyreissi* (described as *Cucujus*), by Perty (1830) for *P. bicolor*, by Guérin-Méneville (1844) for *P. mandibularis* and *P. freyersi* (both noted as *Cucujus*) and by Gray (1832) for *P. dejeani* (described as *Cucujus*) from Brazil (the last four names are recognised as junior synonyms of *P. freyreissi* (von Heyden, [1827]) (Hetschko 1930) and by Waterhouse (1880) for *P. nigriceps* and *P. tenuicornis* from Ecuador. More recently, Jin et al. (2020) described one new genus, *Thesaurus*, with three species; one from Peru (*Thesaurus zaitsevi*), one from Venezuela (*T. albertalleni*) and one from Ecuador (*T. macclarini*). Two species of *Palaestes*: *P. nigriceps* Waterhouse, 1880 and *P. tenuicornis* Waterhouse, 1880, were erroneously recorded from Peru by Blackwelder (1945) in his checklist of South American beetles. The *loci typici* (and the only currently known locations) for these species are in Ecuador, from Chiguinda and Sarayacu [Sarayaku], respectively, as stated by Waterhouse (1880) in the first paragraph of his article. Adding to the confusion, Thomas and Chaboo (2015) noted the occurrence of one genus (presumably *Palaestes*) and three species of Cucujidae in Peru, but later stated that only *P. tenuicornis* was present. Thomas and Chaboo (2015) noted the error in listing the distribution of *P. nigriceps* from Peru by Blackwelder (1945) but, surprisingly, not for his record for *P. tenuicornis*. They also recorded *P. freyreissi* (von Heyden, [1827]) and *P. nigriceps* from South America, but absent from Peru (Thomas and Chaboo 2015). In addition, three Brazilian localities for *P. freyreissi* are published in the GBIF database (www.gbif.org).

**Figure 5.**
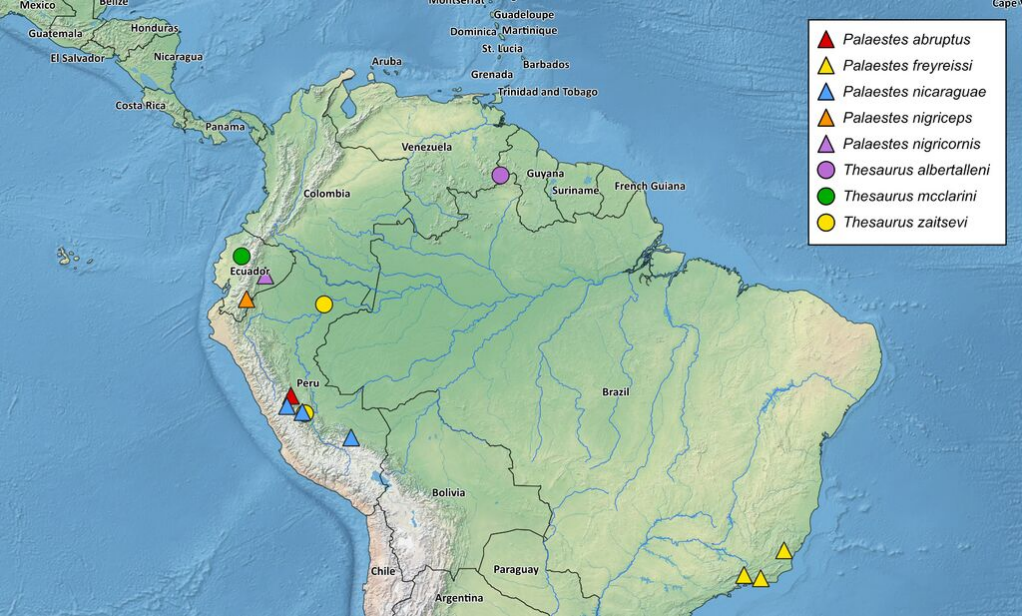
Distribution of Cucujidae in South America.

This paper provides the first records of *P. abruptus* and *P. nicaraguae* from South America, as well as the first confirmed data for the genus *Palaestes* from Peru. Discovery of one of these species was possible thanks to the data collected through the iNaturalist database, which confirms the high value of citizen science in studying biodiversity. Summarising all data mentioned above, we can conclude that the flat bark beetle fauna of South America comprises one subfamily (Platisinae), two genera (*Palaestes* and *Thesaurus*) and at least eight species (Table 1, Fig. 5). Three species are recorded from Ecuador and Peru and one species each from Brazil and Venezuela. Surprisingly, no species have been recorded from the remaining South American countries, despite apparently suitable environments being present in Venezuela, Colombia and Bolivia, at least. Two South American species: *P. nigriceps* Waterhouse, 1880 and *P. tenuicornis* Waterhouse, 1880 from Ecuador have not been recorded from this area since the 19^th^ century, while *P. freyreissi* (von Heyden, [1827]) has not been recorded from Brazil for almost one hundred years.

The two new species records, reported here, highlight the poor state of knowledge of Cucujidae in South America and for the genus *Palaestes* over its wider distributional range (which includes Central America). Most of the published distribution information for *Palaestes* is from the original species descriptions, the most recent of which dates back to 1899. Since that time, the only publications discussing *Palaestes* were reliant on the same locality data as given in the descriptions (viz. Hetschko 1930, Blackwelder 1945). Various entomology collections contain examples of *Palaestes*, which are known to extend the range of distribution records, but these have yet to make it into the scientific literature (Marris, Jaskuła, unpublished data). It is highly likely that further, targeted collecting in appropriate habitats will yield new taxa. This likelihood is emphasised by the recent discovery (the first specimen was collected in 1997) and description of the new genus, *Thesaurus*, with three new species from South America. It is remarkable that, despite more than 220 years of collecting on the continent, these relatively large and distinctive beetles had evaded discovery. Similarly, the state of taxonomy of *Palaestes* species is overdue for revision and there are no keys or modern identification aids available for the genus. The identifications of the two species recorded here are based solely on the original descriptions and photographs of the type material. Examination of existing collection material and detailed, critical analysis of the genus will likely show that new species are present. Both species, recorded here, have been identified as species otherwise known only from localities in Central America, separated by more than 2000 km in a straight line and crossing major geographical barriers. Hopefully, with more emphasis given to surveying for these beetles across Central and South America, more will be revealed about the ecology and biology of both the larvae and adults, as well as providing more information on the range of these distinctive and remarkable beetles.

## Supplementary Material

XML Treatment for Palaestes abruptus

XML Treatment for Palaestes nicaraguae

## Figures and Tables

**Table 1.** Checklist of Cucujidae known from individual countries of South America (countries with no species recorded were not included).

**Species**		**Country**			
		**Brazil**	**Ecuador**	**Peru**	**Venezuela**
1.	*Palaestes abruptus* Sharp, 1899			+	
2.	*Palaestes nicaraguae* Sharp, 1899			+	
3.	*Palaestes freyreissi* (von Heyden, [1827])	+			
4.	*Palaestes nigriceps* Waterhouse, 1880		+		
5.	*Palaestes tenuicornis* Waterhouse, 1880		+		
6.	*Thesaurus albertalleni* Jin, Zwick, Ślipiński, Marris, Thomas & Pang, 2020				+
7.	*Thesaurus macclarini* Jin, Zwick, Ślipiński, Marris, Thomas & Pang, 2020		+		
8.	*Thesaurus zaitsevi* Jin, Zwick, Ślipiński, Marris, Thomas & Pang, 2020			+	
Total		1	3	3	1
